# A history of asthma may be associated with grandparents’ exposures to stress and cigarette smoking

**DOI:** 10.3389/ftox.2023.1253442

**Published:** 2023-09-22

**Authors:** Jean Golding, Holly Tunstall, Steve Gregory, Raquel Granell, James W. Dodd, Yasmin Iles-Caven, Sarah Watkins, Matthew Suderman

**Affiliations:** ^1^ Centre for Academic Child Health, Population Health Sciences, Bristol Medical School, University of Bristol, Bristol, United Kingdom; ^2^ Population Health Sciences, Bristol Medical School, University of Bristol, Bristol, United Kingdom; ^3^ Academic Respiratory Unit, Translational Health Sciences, Bristol Medical School, University of Bristol, Bristol, United Kingdom

**Keywords:** ALSPAC, asthma, childhood stress, childhood smoking, prenatal smoking, grandmother, grandfather, intergenerational

## Abstract

**Introduction:** Within human epidemiological studies, associations have been demonstrated between grandparental exposures during childhood and grandchildren’s outcomes. A few studies have assessed whether asthma has ancestral associations with exposure to cigarette smoking, but results have been mixed so far.

**Material and methods:** In this study we used four generations: (F0 great-grandparents, F1 grandparents, F2 parents, F3 study children) of the Avon Longitudinal Study of Parents and Children (ALSPAC) to determine whether there is evidence of associations between asthma in generations F2 or F3 and exposures to severe trauma in childhood and/or active cigarette smoking during the adolescence of grandmothers and grandfathers in generations F0 and F1 respectively, or of a history of a F0 or F1 grandmother smoking during pregnancy.

**Results:** We have shown that: a) stress exemplified by the death of a F1 grandparent’s parent during the grandparents’ childhood was associated with increased risk of asthma in generation F3, especially if the grandparent involved was the paternal grandmother; b) if the grandparents of generations F0 or F1 smoked during adolescence (i.e. < 17 years), their grandchildren in generations F2 and F3 were more likely to have a history of asthma; c) paternal F1 grandmother’s smoking in pregnancy was associated with her F3 grandchild’s asthma at age 7; d) There were differences between the results for the grandsons and granddaughters of the paternal grandmother with exposure to smoking in adolescence and with smoking in pregnancy. e) The addition of all of the individual exposure variables to the different analyses often provided a considerable increase in goodness of fit compared with only adding demographic factors associated with asthma at *P* < 0.10 such as social class; this was particularly true when all four exposure variables were combined in one model, suggesting possible synergistic effects between them.

**Discussion:** We have shown associations between all four types of exposure to the grandparents to be associated with asthma in the grandchildren, such that the results both depended on whether the male or female line was involved, and the sex of the grandchildren. It was notable that the paternal grandmother was particularly involved in many of the associations. We emphasize that these are exploratory analyses, that asthma diagnostic criteria likely changed over time and may not be consistent between generations, and that the results should be tested in other cohorts.

## Introduction

After exposures to an ancestor in one generation (F0) animal experiments have shown a remarkable pattern of inheritance of different phenotypes from the second generation (F2) onwards. For example, McBirney et al. (2017) showed that exposure of the herbicide atrazine to female F0 rats before breeding compared with controls resulted in differences in phenotype at F2 (their grandchildren) and F3 (their great-grandchildren) but not among the F1 generation (their offspring). The results were particularly striking for multiple disorders in the same animal. These longitudinal associations are not just associated with maternal exposures, but paternal exposures before or during puberty have also been shown to have effects down the generations. Manners et al. (2019), for example, showed that stress to adolescent male rodents prior to mating was associated with abnormalities in the F2 and F3 generations, with phenotypic differences between the sexes.

There are now several observational human studies that have shown associations between exposures in the F0 generation being associated with phenotypic differences in the F2 and F3 generations. These differences down the generations were bolstered by a unique study in Överkalix in the far north of Sweden which identified changes in the grandchildren’s death rate according to whether their grandparents had experienced a famine or an exceptionally good harvest during their childhoods. The data indicated that the associations varied with the sex of the grandchild and according to whether the grandmother or grandfather was exposed (Pembrey et al., 2006); the main findings concerning exposure to a good harvest were validated by a later study in Sweden (Vågerö et al., 2018). Other studies that showed associations between exposures to the F0 generation in childhood and outcomes in the F2 generation concerned exposures to a severe famine in Germany during World War I (van den Berg and Pinger, 2016). The authors showed that the grandparents who had been exposed during their pre-puberty (slow growth) period had grandchildren who had better mental health than expected. Although famine appeared to be the exposure of interest, the data could equally be interpreted as an exposure to a traumatic event. To assess whether similar associations between an ancestral exposure and a variety of outcomes are apparent nowadays, several longitudinal studies have used both ancestral smoking and stressful events experienced in childhood by grandparents and examined the associations with outcomes of the grandchildren and great-grandchildren (e.g., Vågerö et al., 2018; Manners et al., 2019; Golding et al., 2021; Gregory et al., 2022; Golding et al., 2023).

In this paper we concentrate on associations between: a) grandparental smoking in adolescence, b) grandmother smoking during pregnancy, or c) grandparental exposure to a severe trauma (the death of a parent) during their childhoods, and asthma in their grandchildren. There have been five previous longitudinal and one case-control study which considered smoking of the *maternal* grandmother during pregnancy and asthma or persistent wheezing in the grandchild: these showed mixed results and little consistency (Table 1). Some studies showed associations with grandchild’s asthma, some with wheeze, one indicated an increased risk to the grandchild who wheezed persistently, one only if the mother and the grandmother had both smoked. Only two studies had considered whether the *paternal* grandmother had smoked during the pregnancy that resulted in the father’s birth, one of these showed an increase in clinically diagnosed asthma in the grandchild but no such association with *maternal* grandmother smoking. It is notable that the definitions of asthma differed between the various studies, as did the ages of the children when the prevalence of asthma was recorded.

**TABLE 1:** Published results from longitudinal human studies concerning exposures to cigarette smoking in grandmothers and asthma and wheezing in their grandchildren. Associations at *p* < 0.05 are in bold.

Author	Country (no. Grandchildren)	Ancestors	Exposure	Outcomes	Prev	Results MGM	Results PGM	Comment
		Exposed	Smoking	Considered				
POPULATION STUDIES of ASTHMA								
Bråbäck et al. (2018)	Sweden (10,329)	MGMPGM	earlypreg	Asthma^a^ at 6y		AOR [95%CI]	AOR [95%CI]	No difference between sexes
				ETA	2.6%	.99 [.80,1.22]	1.10 [.90,1.36]	
				EPA	4.3%	**1.27[1.08,1.49]**	.93 [.79,1.09]	
				LOA	3.5%	.97 [.80,1.19]	.91 [.75,1.11]	
Magnus et al. (2015)	Norway (53,169; 25,394; 45,607)	MGM	Preg	Asthma^b^		ARR [95%CI]	-	No difference between sexes
				at 3 years	5.7%	**1.15[1.06,1.24]**		
				at 7 years	5.1%	**1.21[1.07,1.37]**		
				Link^a^ at 7y	4.8%	**1.15[1.04,1.26]**		
Mahon et al. (2021)	Netherlands (11,544; 25,747)	MGM	Preg	Children^b^		AOR [95%CI]		
				at 4years	8.3%	.92 [0.78,1.09]		
				<18 years^b^	11.4%	.88 [0.76,1.03]		
				Adults				
				- at 4 years	4.4%	**1.35[1.06,1.71]**		
				- >17 years	11.0%	**1.18[1.00,1.39]**		
Miller et al. (2014)	United Kingdom	MGM	Preg	Dr. Diagnosed asthma at 7	10.5%	AOR^c^	AOR [95%CI]	Sex interaction for PGM: *p* = .111
	Avon (12,506)	PGM		Persistentwheezing		1.01 [.84,1.22]	1.17 [.97,1.41]	
						1.26 [.95,1.67]	Girls	
							**1.39[1.04,1.86]**	
							1.08 [.79,1.47]	
Arshad et al. (2017)	United Kingdom	MGM	Preg	Wheeze	?	OR^c^		If MGM + M
	Isle of Wight					.90 [0.3, 2.4]		OR = 2.6 [.90, 7.1]
CASE—CONTROL STUDY								
Li et al. (2005)	United States of America	MGM	Preg	Asthma at 5		**2.1[1.4, 3.2]**		MGM + M
	338 (with asthma, 570 control)							

^a^ Identified from linked prescriptions for corticosteroids.

^b^ Asked asthma in questionnaires.

^c^ Excludes mothers who smoked in pregnancy.

Key: ALSPAC, Avon Longitudinal Study of Parents and Children; AOR, adjusted odds ratio; ARR, adjusted relative risk; CI, confidence interval; EPA, early persistent asthma; ETA, early transient asthma; LOA, late onset asthma; MGM, maternal grandmother; PGM, paternal grandmother; OR, odds ratio; Preg = in pregnancy; Prev = prevalence; Qs = Questionnaires.

No one, to our knowledge, has examined whether a grandchild’s respiratory outcomes are associated with grandparental traumas during their childhoods or with grandparents’ cigarette smoking during their adolescence (which has been shown to be associated with increased fat mass in the grandchildren, especially when the paternal rather than the maternal grandfather is involved) (Gregory et al., 2022).

The aims of this study, therefore, are:

i) To extend the previous analyses (Miller et al., 2014) from the Avon Longitudinal Study of Parents and Children (ALSPAC) to look at the grandparental associations with smoking in pregnancy using different ages for which the prevalence of asthma was recorded, and different generations (i.e., the adult grandchildren (F2) and the grandchildren at ages 7 and 22 (F3)), in regard to exposures to their grandparents in generations F0 and F1 respectively, and thus to determine whether associations changed with age (F3) or over time (results for F2 compared with results for F3).
ii) In line with our other recent inter/trans-generational studies (Golding et al., 2023), we will also assess whether traumatic events occurring in childhood or smoking of the grandparent during adolescence (Gregory et al., 2022) are associated with asthma in the grandchild.

## Materials and methods

**FIGURE 1:**
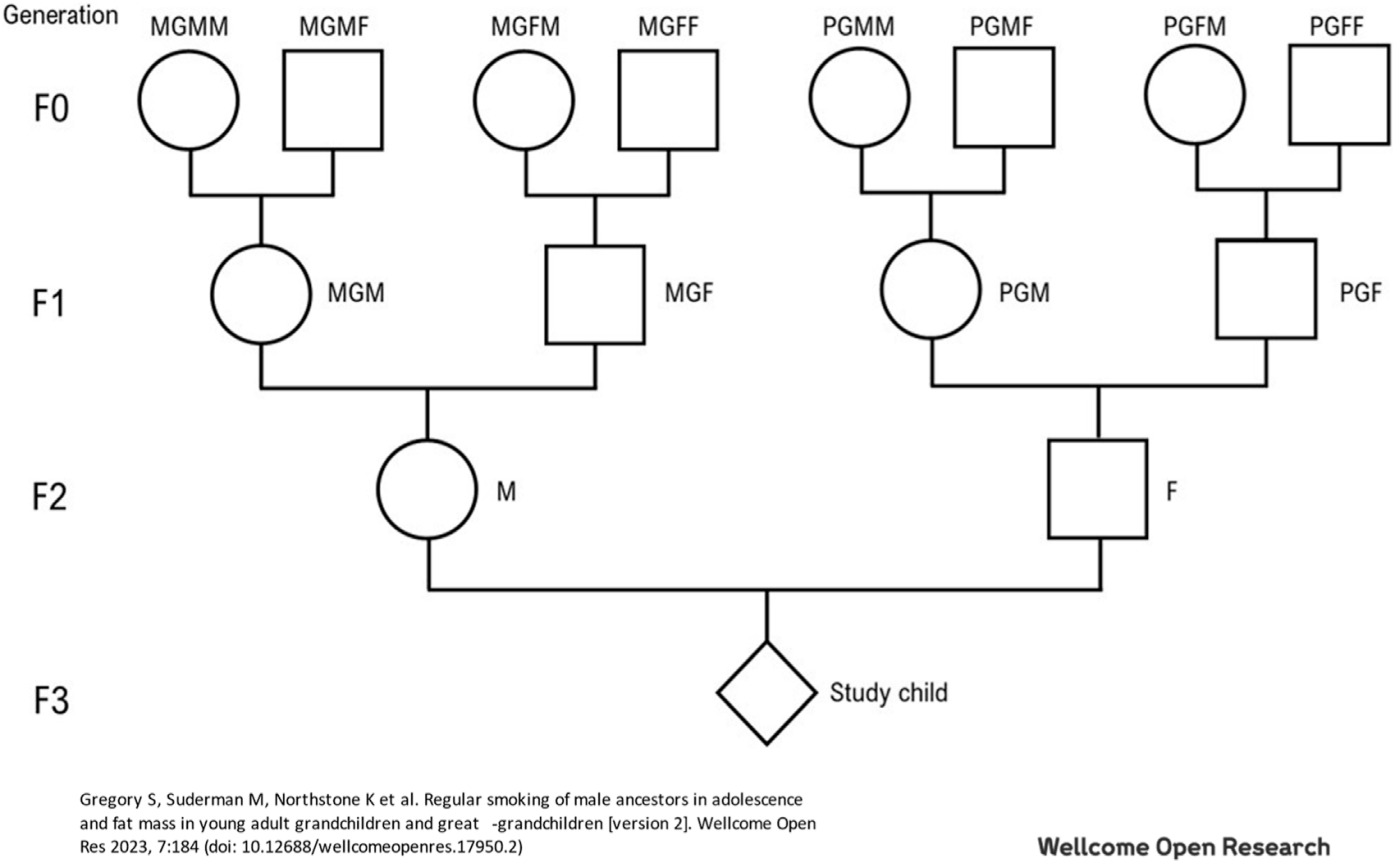
Family Structure with nomenclature used (see text in Methods section).

Unlike experimental studies which start with an F0 proband and progress down the generations, we start with a proband in the F2 (or F3) generation and work backwards to the exposures to members of the F0 (or F1) generation. This results in the study of four possible lines of inheritance from grandparents to grandchildren (Figure 1). We compare results between the paternal and maternal lines, as well as determining whether there are sex differences between outcomes in the grandchildren.

### The ALSPAC cohorts

The initial ALSPAC cohort was defined as all offspring of women who had been resident in a defined area (known as Avon) during pregnancy, and whose expected date of delivery lay between 1 April 1991 and 31 December 1992. The number of pregnancies enrolled was 14,541. Of these, there was a total of 14,676 fetuses, resulting in 14,062 live births and 13,988 children who were alive at 1 year of age. Subsequent attempts to enrol eligible women who had not initially enrolled resulted in a total sample size for analyses (including data collected after the age of seven) of 15,447 pregnancies, resulting in 15,658 fetuses. Of these, 14,901 children were alive at 1 year of age. (Boyd et al., 2013; Fraser et al., 2013; Northstone et al., 2019). From the F3’s age of 22, study data have been collected and managed using REDCap electronic data capture tools hosted at the University of Bristol. REDCap (Research Electronic Data Capture) is a secure, web-based software platform designed to support data capture for research studies (Harris et al., 2009). The ALSPAC website contains details of all the data that are available through a fully searchable data dictionary and variable search tool: http://www.bristol.ac.uk/alspac/researchers/our-data/

Ethical approval for the study was obtained from the ALSPAC Ethics and Law Committee (ALEC; IRB00003312) and the Local NHS Research Ethics Committees. Detailed information on the ways in which confidentiality of the cohort is maintained may be found on the study website: http://www.bristol.ac.uk/alspac/researchers/research-ethics/. All methods were performed in accordance with the relevant guidelines and regulations at the time. Informed consent for the use of data collected via questionnaires and clinics was obtained from participants following the recommendations of the ALSPAC Ethics and Law Committee at the time (Birmingham, 2018).

### Data collection methods

Information on the offspring cohort was collected using a variety of methodologies, including: a) self-completion questionnaires sent to the parents, the child him/herself and the child’s teachers; b) hands on examinations in a clinical setting at various ages; c) linkage to various sources of information including educational and medical records.

### Information collected on the ancestors

As noted previously, the aim of this study was to consider the environmental exposures to the ancestral generations. Consequently, questionnaires were administered to the ALSPAC parents concerning their own parents and grandparents using self-completion questionnaires which they received by mail. The recipients were encouraged to enquire of other family members if they did not know the answers to specific questions. The first set of such questions concerning the study grandparents was asked during the index pregnancies and included data on educational achievements and occupations, their ages at birth of their offspring as well as basic questions concerning their health and smoking habits. As previously noted in Golding et al. (2020): ‘The data collected on the study child’s grandparents and great grandparents comprised: a) countries of birth; b) years of birth; c) age at onset of smoking; d) whether the ancestral mothers smoked during pregnancy; e) occupation (from which the social class was derived); f) information on 19 potentially traumatic situations in their childhoods such as death of a parent, being taken into care, not having enough to eat, or being in a war situation; g) causes of death for those ancestors who had died. The ages at which the individual experienced the traumatic situations distinguished between ages <6, 6–11, and 12–16 years. (see Golding et al., 2020 for full details of the variables and their frequencies)’.

**FIGURE 2:**
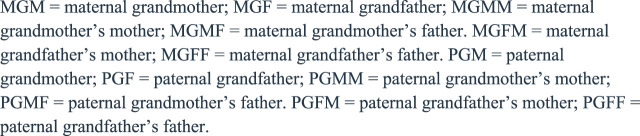
Abbreviations used.

Figure 1 shows the relationships between the generations. It is used in these analyses to identify two sets of relationships between grandparents and grandchildren: i) between generations F1 and F3, and ii) between generations F0 and F2. For example, using the nomenclature in Figure 1 and Figure 2, the maternal grandmother of the study child is MGM, the maternal grandmother of the study father is PGMM.

### Statistical analyses

This is an exploratory set of analyses to determine whether there are any indications that the onset of asthma may be linked to ancestral exposures. We examine whether there is evidence that grandparents’ exposures to smoking or to traumatic events such as the death of a parent are related to asthma in their grandchildren. Because of its exploratory nature, we reduced the chance of Type I errors by considering associations at *p* < 0.05 to be of interest even if they did not pass the Bonferroni adjusted threshold of *p* = 0.05/50 = 0.001. It is important to recognise that such evidence is exploratory and requires other confirmatory data before assuming the associations to be robust.

Numbers of individuals available for analysis in relation to asthma at age 7 are shown in Supplementary Table S1. The numbers of granddaughters with data available was greater than the numbers of grandsons; the numbers relating to the maternal grandparents were greater than those relating to the paternal grandparents and, apart from social class, the numbers available for other features were greater for grandmothers than grandfathers. Consequently, the statistical power was greatest for the maternal grandparents of the granddaughters.

The statistical analyses used logistic regression with a history of asthma as the binary outcome. We considered all available demographic variables related to the ancestor to be potential confounders if their unadjusted relationship with the outcome was *p* < 0.10. The model for each set of such confounders for each ancestor/outcome data set was called Model A. We used pseudo-R^2^ as an estimate of goodness of fit (GoF) and examined the change in GoF with addition of each environmental exposure to Model A. The first step in the analysis of each environmental exposure was to insert these potential confounders into the unadjusted regression and record the adjusted odds ratio (AOR) and 95% confidence interval (CI) together with the GoF. Finally, all environmental exposures to the specific ancestor were inserted together with the factors in Model A, to form Model B, and the difference in GoF was recorded.

To identify differences between the sexes we compared the pairs of relevant adjusted odds ratios—if these were each outside the 95% CIs of the opposite sex, it was assumed that they differed from one another.

Analyses were repeated for grandchildren using the F0 to F2 and F1 to F3 generations. For the F2 generation (the mothers and fathers of the study child) we conducted the analyses for each parent with the outcome as to whether they had ever had asthma; separate analyses were repeated for the male and female F3 grandchildren at ages 7 and 22.

### Environmental exposures considered

We tested three ancestral exposures to each of four grandparent-grandchild sequences (F0—F2; F1—F3) and the smoking in pregnancy exposure to the two grandmothers (F0—F2; F1—F3). All data were collected using self-completion questionnaires sent to the study parents and their adult offspring (generations F2 and F3):

i) Smoking during the grandmothers’ pregnancy (binary variable; collected during the enrolment pregnancy for the F1 generation;
ii) Smoking regularly during the F0 and F1 grandparents’ adolescence (before age 17);
iii) Death of the F0 and F1 grandparent’s mother before age 17;
iv) Death of the F0 and F1 grandparent’s father before age 17.

The prevalence’s of these exposures among the different generations are shown in Table 2. It can be seen that, in general, the proportions of grandparents who had lost a father during childhood was considerably greater than the proportion who had lost their mother; and that the proportion of grandfathers who had started smoking regularly during childhood was greater (42%–46%) than for grandmothers (17%–23%).

**TABLE 2:** The proportion of the grandparents with information on asthma that had experienced the environmental exposures (in brackets are the numbers who had such an experience).

Grandparent	Mother died in childhood	Father died in childhood	Smoked in childhood	Smoked during pregnancy
*Generation F3 At Age 7*				
MGM	9.7% (346)	15.7% (540)	17.8% (575)	34.7% (2647)
MGF	9.7% (328)	17.1% (562)	45.3% (1279)	-
PGM	10.5% (196)	18.5% (294)	17.4% (250)	23.2% (315)
PGF	13.2% (210)	18.4% (287)	42.1% (523)	-
*Generation F2 Women Aged 14–47*				
MGM	9.9% (376)	16.8% (612)	17.9% (617)	33.5% (3132)
MGF	9.7% (350)	16.9% (592)	45.8% (1385)	-
PGM	12.1% (213)	18.1% (304)	17.3% (263)	33.8% (3749)
PGF	13.3% (223)	18.6% (306)	42.1% (557)	-
*Generation F2 Men Aged 15–65*				
MGM	9.8% (289)	16.1% (455)	19.2% (512)	36.0% (2847)
MGF	9.8% (275)	16.7% (454)	45.6% (1078)	-
PGM	11.4% (181)	17.1% (258)	22.7% (293)	41.2% (3114)
PGF	12.5% (190)	17.9% (265)	41.0% (488)	-

MGM, maternal grandmother; MGF, maternal grandfather; PGM, paternal grandmother; PGF, paternal grandfather.

### Outcome measures

There were four asthma outcome measures:

1) At age 7 of the F3 generation the question answered by the mother was: ‘Has a doctor ever actually said that your child has asthma?’, 20.4% of 8168 responses were positive.
2) At age 22, the question asked of the grandchild (F3) was: ‘have you ever had asthma?’ 24.9% replied positively.
3) For asthma in the F2 generation, the question asked of the study mother and her partner (referred to henceforth as the father) was: ‘have you ever had asthma?’. Positive replies were received by 11.3% of mothers (at ages 14–47) and 12.8% of fathers (at ages 15–65).

### Potential confounders considered

The following features of the grandparents and great-grandparents were considered as potential confounders:

a) Year of birth: (treated as a continuous variable)
b) Age at the birth of the subsequent generation (i.e., for grandparents of the F2 generation, it was the age they were at the time of birth of the relevant F1 generation; for the F1 grandparents of the F3 generation it was their age at the time that the relevant F2 parent was born).
c) Place of birth: compared births to residents of England with elsewhere in the world.
d) Education level: was based on the highest qualification obtained and compared those with the equivalent of the United Kingdom O-level (16-year-old qualification) or greater with less than O-level. This information was not available for the F0 generation.
e) Social class: was based on occupation; for the F1 grandparents, six classes were used graded from professional to unskilled manual occupations, and for the F0 grandparents, non-manual occupations were compared with manual occupations.

The prevalence of asthma with each of these variables was identified as a confounder if the association was at *p* < 0.05. The relationships between the demographic variables associated with the grandparents and each of the four outcomes is shown in Supplementary Tables S2A–D.

## Results

### Exposures to the maternal grandmothers

Maternal grandmothers are represented twice in the Family Tree (Figure 1): in generation F0, MGMM is the maternal grandmother of the study mother (M) in generation F2; and in generation F1, MGM is the maternal grandmother of the study child (generation F3). Unadjusted and adjusted associations between the four environmental exposures to the maternal grandmother and asthma in her grandchild are shown in Table 3. For F3 asthma at 7 years there were unadjusted associations at *p* < 0.05 for three of the four environmental exposures, including the two measures of stress in childhood measured by death of mother and father. On adjustment, only the exposure to the death of the mother retained significance: [AOR 1.46; 95%CI 1.01, 2.11]. No such associations were found for the F3 asthma at 22 years measure or the F2 measures.

**TABLE 3:** The associations between the exposures to the ***maternal grandmothers* and asthma in the grandchildren, where Model A comprises all the confounders, and Model B comprises all confounders together with the exposures. Associations at *p* < 0.05 are in bold.**

*F3. Asthma age 7 years*	Unadjusted			Adjusted			GoF	Change in GoF
	OR [95%CI]	P	N	OR [95%CI]	P	N		
MODEL A^a^ Exposures						1989	.54	
Death of mother	**1.46[1.12,1.90]**	**.005**	3574	**1.46[1.01,2.11]**	**.046**	1963	.72	+33%
Death of father	**1.26[1.01,1.58]**	**.044**	3434	1.33 [.98,1.78]	.063	1894	.72	+33%
Smoked in adolescence	1.22 [.97,1.52]	.084	3226	1.25 [.92,1.70]	.154	1760	.87	+56%
Smoked during pregnancy	**1.16[1.03,1.30]**	**.012**	7627	.94 [.73,1.21]	.634	1915	.46	−15%
MODEL B						1623	1.16	+115%
***F3. Asthma age 22 years***								
MODEL A^b^						2219	.06	
Death of mother	1.07 [.79, 1.46]	.660	2389	.90 [.65, 1.26]	.551	2179	.10	+67%
Death of father	1.10 [,85, 1.43]	.454	2294	.90 [.69, 1.19]	.461	2089	.11	+83%
Smoked in adolescence	1.11 [.85, 1.44]	.448	2169	1.11 [.84, 1.46]	.473	1979	.11	+83%
Smoked during pregnancy	1.04 [.88, 1.22]	.667	3515	.97 [.78, 1.21]	.765	2116	.04	−33%
MODEL B						1796	.18	+200%
***F2. Asthma women aged 14–47***								
MODEL A^3^							.00	
Death of mother	.88 [.60,1.29]	.509	1196	.85 [.56,1.29]	.444	1905	.05	
Death of father	.88 [.61,1.28]	.504	1052	1.02 [.69,1.51]	.921	1685	<.01	
Smoked in adolescence	1.09 [.77,1.54]	.632	1133	1.17 [.81,1.69]	.414	1821	.05	
Smoked during pregnancy	1.05 [.69,1.60]	.811	965	1.44 [.96,2.16]	.075	1535	.27	
MODEL B						908	.47	
***F2. Asthma Men aged 15–65***								
MODEL A^4^							.00	
Death of mother	1.13 [.58,2.20]	.713	605	1.08 [.56,2.09]	.816	814	.01	
Death of father	1.41 [.74,2.67]	.292	537	1.40 [.74,2.65]	.298	543	.24	
Smoked in adolescence	.67 [.33,1.35]	.259	582	.65 [.32,1.31]	.225	588	.34	
Smoked during pregnancy	.94 [.82, 1.08]	.389	7919	.65 [.27, 1.56]	.333	503	.34	
MODEL B						268	1.35	

^a^ Confounders were year of birth, age and resident in England.

^b^ Confounders were year of birth and residence in England.

^3,4^There were no confounders.

### Exposures to the maternal grandfathers

Maternal grandfathers are represented twice in the Family Tree (Figure 1): MGMF in generation F0 is the maternal grandfather of the study mother (M) in generation F2; and MGF in generation F1 is the maternal grandfather of the study child in generation F3. Unadjusted and adjusted associations between the four environmental exposures to the maternal grandfather and asthma in his grandchild are shown in Table 4. For F3 grandchildren there was only one unadjusted association at *p* < 0.05 for an association between death of MGF’s mother during his childhood and his grandchild’s asthma at age 7: [OR 1.37 (95%CI 1.04, 1.80)]; upon adjustment the OR hardly changed although the confidence interval was wider because of smaller numbers [AOR 1.30 (95% CI 0.90,1.87)].

**TABLE 4:** The associations between the exposures to the ***maternal grandfathers* and asthma in the grandchildren, where Model A comprises all the confounders, and Model B comprises all confounders together with all the exposures. Associations at *p* < 0.05 are in bold.**

Exposure	Unadjusted			Adjusted			GoF	%Change in GoF
	OR [95%CI]	P	N	OR [95%CI]	P	N		
***F3. Asthma age 7 years***								
MODEL A^1^						4310	.38	
Death of mother	**1.37[1.04,1.80]**	**.025**	3388	1.30 [.90,1.87]	.166	2132	.94	+141%
Death of father	1.17 [.93,1.47]	.177	3277	.91 [.66,1.26]	.583	2073	.84	+121%
Smoked in adolescence	1.04 [.86,1.26]	.868	2826	.99 [.76,1.29]	.958	1785	.92	+142%
MODEL B						1123	.62	+63%
***F3. Asthma age 22 years***								
MODEL A^2^						2125	.19	
Death of mother	1.17 [.85,1.59]	.340	2271	1.28 [.84,1.93]	.248	1397	.34	+79%
Death of father	.89 [.67,1.17]	.396	2192	.70 [.47,1.04]	.081	1357	.55	+19%
Smoked in adolescence	1.07 [.86,1.32]	.547	1925	.97 [.73,1.30]	.847	1188	.37	+95%
MODEL B						1123	.62	+226%
***F2. Asthma women aged 14–47***								
MODEL A^3^							.00	
Death of mother	.83 [.53,1.30]	.423	898	1.20 [.76,1.89]	.437	1416	.06	
Death of father	.90 [.57,1.41]	.645	833	1.25 [.80,1.96]	.324	1304	.10	
Smoked in adolescence	.87 [.63,1.21]	.419	779	1.02 [.72,1.44]	.916	1260	<.01	
MODEL B						708	.27	
***F2. Asthma men aged 15–65***								
MODEL A^4^							.00	
Death of mother	.76 [.33,1.75]	.518	459	.77 [.33,1.77]	.536	463	.11	
Death of father	1.31 [.64,2.65]	.461	465	1.29 [.64,2.62]	.477	469	.13	
Smoked in adolescence	1.18 [.68,2.05]	.554	448	1.22 [.71,2.11]	.477	452	.14	
MODEL B							.75	

^a^ Year of birth, age and education.

^b^ Year of birth and age.

^3,4^There were no confounders.

### Exposures to the paternal grandmothers

Paternal grandmothers are represented on three occasions in the Family Tree (Figure 1): MGFM in the F0 generation is the paternal grandmother of the study mother (M) in F2; similarly, PGFM in F0 is the paternal grandmother of the study father (F) in F2; and PGM in the F1 generation is the paternal grandmother of the study child in F3.

Unadjusted and adjusted associations at *p* < 0.05 were found between asthma of the F3 grandchild and PGM’s death of the father during her childhood, her smoking in adolescence and smoking in pregnancy. There were no such associations when these grandchildren were aged 22. (Table 5). However, the F2 males (i.e., grandsons of their PGMs) showed an increase in asthma prevalence when their paternal grandmother had smoked in adolescence [AOR = 2.64 (95%CI 1.26, 5.54)].

**TABLE 5:** The associations between the exposures to the ***paternal grandmothers* and asthma in the grandchildren, where Model A comprises all the confounders, and Model B comprises all confounders together with all the exposures. Associations at *p* < 0.05 are in bold.**

Exposure	Unadjusted			Adjusted			GoF	%Change in GoF
	OR [95%CI]	P	N	OR [95%CI]	P	N		
***F3. Asthma age 7 years***								
MODEL A^a^							.49	
Death of mother	1.27 [.88,1.85]	.204	1664	1.30 [.76,2.23]	.334	991	.55	+12%
Death of father	**1.52[1.10,2.09]**	**.010**	1587	**1.60[1.03,2.48]**	**.037**	953	.95	+94%
Smoked in adolescence	**1.47[1.05,2.07]**	**.026**	1436	**1.89[1.21,2.97]**	**.006**	856	1.45	+194%
Smoked during pregnancy	**1.26[1.11,1.43]**	**<.001**	6231	**1.31[1.10,1.56]**	**.003**	3319	.77	+57%
MODEL B						797	2.41	+392%
***F3. Asthma age 22 years***								
MODEL A^2^							.00	
Death of mother	.93 [.61,1.41]	.720	1244	.93 [.61,1.41]	.726	1244	.01	
Death of father	1.19 [.85,1.67]	.313	1189	1.19 [.85,1.67]	.313	1189	.08	
Smoked in adolescence	1.24 [.85,1.81]	.260	1078	1.24 [.85,1.81]	.260	1078	.11	
Smoked during pregnancy	1.16 [.98,1.38]	.081	2951	1.16 [.98,1.38]	.081	2951	.09	
MODEL B						881	.39	
***F2. Asthma women aged 14–47***								
MODEL A						1237	.75	
Death of mother	.85 [.53,1.35]	.492	782	.98 [.54,1.76]	.938	788	1.82	+156%
Death of father	.83 [.51,1.36]	.466	714	1.64 [.97,2.78]	.067	727	2.27	+203%
Smoked in adolescence	.85 [.53,1.35]	.488	784	.90 [.47,1.70]	.737	738	1.15	+53%
Smoked during pregnancy	1.13 [.67,1.91]	.644	658	.69 [.32,1.50]	.348	619	1.36	+81%
MODEL B						362	6.01	+701%
***F2. Asthma Men aged 15–65***								
MODEL A						568	.69	
Death of mother	1.00 [.45,2.24]	.991	429	1.02 [.98,1.06]	.917	309	.28	−63%
Death of father	.50 [.19,1.31]	.160	399	.25 [.06,1.10]	.067	290	2.56	+271%
Smoked in adolescence	**2.15[1.19,3.87]**	**.011**	451	**2.64[1.26,5.54]**	**.010**	291	2.39	+246%
Smoked during pregnancy	1.22 [.51,2.91]	.655	390	1.37 [.45,4.20]	.578	249	.67	−3%
MODEL B						133	2.50	+525%

^a^ Year of birth, age and education.

^b^ Age.

^2,4^There were no confounders.

### Exposures to the paternal grandfathers

As with other grandparents, the paternal grandfathers are represented three times in the Family Tree (Figure 1): MGFF in generation F0 is the paternal grandfather of the study mother (M) in generation F2, PGFF in generation F0 is the paternal grandfather of the study father (F) in generation F2 and PGF in generation F1 is the paternal grandfather of the study child in generation F3. There were no unadjusted or adjusted associations at *p* < 0.05 between the environmental exposures to the paternal grandfathers and increased risk of asthma in their grandchildren. (Table 6).

**TABLE 6:** The associations between the exposures to the *paternal grandfathers* and asthma in the grandchildren, where Model A comprises all the confounders, and Model B comprises all confounders together with the exposures.

Exposure	Unadjusted			Adjusted			GoF	%Change in GoF
	OR [95%CI]	P	N	OR [95%CI]	P	N		
***F3. Asthma age 7 years***								
MODEL A^1^						3104	.49	
Death of mother	1.08 [.74,1.58]	.680	1585	1.41 [.84,2.36]	.198	913	.49	0%
Death of father	1.24 [.89,1.72]	.198	1553	1.51 [.98,2.34]	.060	900	.76	+55%
Smoked in adolescence	1.22 [.91,1.63]	.192	1242	1.47 [.96,2.25]	.075	719	1.22	+149%
MODEL B						681	1.37	180%
***F3. Asthma age 22 years***								
MODEL A^2^							.00	
Death of mother	.86 [.57,1.28]	.453	1183	.86 [.57,1.28]	.453	1183	.04	
Death of father	1.19 [.85,1.68]	.312	1156	1.19 [.85,1.68]	.312	1156	.08	
Smoked in adolescence	1.27 [.93,1.73]	.128	936	1.27 [.93,1.73]	.128	936	.23	
MODEL B						840	.21	
***F2. Asthma women aged 14–47***								
MODEL A						848	2.05	
Death of mother	.91 [.50,1.65]	.746	658	.68 [.23,1.99]	.483	557	2.23	+9%
Death of father	1.62 [.97,2.70]	.065	630	1.69 [.82,3.48]	.151	538	3.20	+56%
Smoked in adolescence	1.09 [.73,1.61]	.678	573	1.27 [.68,2.37]	.460	406	2.20	+7%
MODEL B						269	6.95	+219%
***F2. Asthma Men aged 15–65***								
MODEL A							.00	
Death of mother	1.13 [.58, 2.20]	.713	605	1.38 [.58,3.28]	.470	400	.16	
Death of father	1.41 [.74,2.67]	.292	537	.43 [.15,1.25]	.122	417	.94	+
Smoked in adolescence	.67 [.33,1.35]	.259	582	1.16 [.63,2.15]	.637	346	.08	-
MODEL B						167	1.12	+

^a^ Year of birth, education, age and social class.

^b^ Year of birth and age.

^2,4^There were no confounders.

### Differences in goodness of fit

In each of Tables 3, Tables 4, Tables 5, Tables 6 the % changes in GoF are given when an environmental exposure is added to Model A for those sets of analyses where confounders were appropriate. However, this was not a judgement that could be made if there were no variables in Model A. Among the analyses where model A had a GoF >0, the analysis of Model B resulted in increases of at least 200% in six instances: one each for MGM, MGF and PGF, but three for PGM where the addition of all four exposures resulted in increases in GoF of 392% for the F3 grandchildren at age 7; for the F2 male grandchildren it was 701%, and for the F2 female grandchildren it was 525%.

### Differences between the sexes

Similar analyses were carried out separately for the two F3 sexes at ages 7 and 22 (Supplementary Tables S3A, B). Adjusted associations at *p* < 0.05 were found for five results for the grandchildren at age 7 (two with grandsons, three with granddaughters); four of the five were associated with the paternal grandmother. At age 22 just two of the AORs were at *p* < 0.05, (one (mother died) with grandsons [AOR .44 (95%CI .23, .84)] and one (father died) with granddaughters [AOR .58 (95%CI .35, .96)]). However, there were three instances where the 95% CIs indicated differences between the two (Supplementary Tables S3A, B): i) among 7-year-old grandchildren whose paternal grandmother had smoked in adolescence, the AOR was 2.89 (95%CI 1.57, 5.35) for grandsons developing asthma compared with 1.18 95%CI 0.59, 2.39) for the granddaughters; ii) at 22 years, if the paternal grandmother had smoked in pregnancy the grandsons were less likely to have a history of asthma [(AOR = 0.90 (95%CI .67,1.20)] than the granddaughters [(AOR = 1.20 (95%CI .97, 1.33); iii) if the maternal grandfather’s mother had died during his childhood, his 22-year-old grandsons were even less likely to have a history of asthma [AOR .44 (95%CI .23,.84) than his granddaughters [AOR 0.89 (95%CI .53,1.54).

### Summary of relationships between grandparent exposures and asthma in the grandchildren

Excluding the sex-specific analyses of the F1 grandchildren, there were 56 separate analyses. Of these five of the AORs were at *p* < 0.05, no more than the 2.8 expected by chance. Of the 56 sex specific analyses of the F3 grandchildren, there were seven AORs at *p* < 0.05, compared with 2.8 expected [chi-squared = 6.3; *p* < 0.05].

It can be seen from the Tables (including the Supplementary Tables) that after adjustment, the following relationships between grandparent exposures and asthma were apparent at *p* < 0.05:

#### Stress in childhood

There was a difference between asthma in the F2 adult women at ages 14–47 and the F2 adult men at ages 15–65: if their G0 paternal grandmother had experienced the death of their father, the AOR suggested a protective effect for the adult men [AOR 0.25 (95%CI .06, 1.10)] in contrast to the adult women [AOR = 1.64 (95%CI .97,2.78)].

#### Smoking in adolescence

An increased likelihood of childhood asthma was found among both 7-year-olds in generation F3 and adult men in generation F2 if their paternal grandmother had smoked regularly in adolescence [AOR = 1.89 (95%CI .21,2.97)] and [AOR 2.64 (95%CI 1.26,5.54) respectively); this was particularly true of their grandsons [AOR 2.89 (95%CI 1.59, 5.35) compared with their granddaughters (AOR 1.18 (95%CI .59,2.39). There were no such associations if their maternal grandmother had behaved in this way, nor were there any adjusted associations at *p* < 0.05 when the grandfathers had smoked in adolescence.

#### Smoking in pregnancy

There were no adjusted associations at *p* < 0.05 with the *maternal* grandmother smoking in pregnancy.; However, generation F1 *paternal* grandmother smoking was associated with generation F3 grandchildren having increased risk of a history of asthma at age 7 [AORs 1.31 (95%CI 1.10,1.56)]), with similar risks for grandsons [AOR 1.30 (95%CI 1.02, 1.64)] and granddaughters [AOR 1.33 (95%CI 1.02, 1.73).

## Discussion

In the past there has been some evidence that grandmothers’ smoking in pregnancy may be associated with an increase in the prevalence of asthma in their grandchildren. In this set of analyses of multigenerational data in ALSPAC we have addressed the question as to whether that is true for different ages and different generations, and whether grandparental exposures during their childhoods to either stress or to active onset of regular cigarette smoking may be linked to asthma in their grandchildren. Numbers available for analysis were limited, so the analyses shown are aimed at identifying possible associations—to be tested in other intergenerational cohorts. We have shown that: a) stress exemplified by the death of a F0 or F1 grandparent’s father or mother during his/her childhood was associated with increased risk of asthma in generation F3; b) Onset of smoking in adolescence of generations F0 and F1 grandparents was associated with history of asthma in generations F2 and F3; c) paternal grandmother smoking in pregnancy was associated with history of asthma at age 7 of the F3 generation; d) goodness of fit (GoF) comparisons showed that the addition of many of the individual exposure variables often provided a substantial increase in GoF which was not apparent for the individual exposure variables implying possible synergistic effects between these environmental exposures. This increase was especially marked for exposures to the paternal grandmother.

It is notable that, of the demographic factors relating to the ancestors, the variable most associated with asthma in the grandchild was the grandparent’s year of birth. It is not surprising that this was linked to cigarette smoking since in the mid-20th century in England, the prevalence of smoking among the 25–34-year age group was estimated to be as high as 80% among men and 53% among women (Peto et al., 2000). Consequently, a large proportion of the grandparents in this study will have been smokers. However, there is less information on the proportion taking up the habit during childhood within the United Kingdom. In comparing the cigarette smoking between the generations, it is important to remember that the types and constituents of the commonly smoked cigarettes changed over time. The fact that we found largely similar results between the generations for intergenerational associations with grandparents’ smoking in adolescence may indicate that, if this is a causal relationship, the constituents of cigarettes that changed over time may not be responsible for the findings.

Although animal studies of intergenerational and transgenerational effects are well documented. It is acknowledged that multigenerational human studies with sufficient data on exposures and health outcomes are rare (Arshad et al., 2017). Published studies have mainly concentrated on ancestral exposures to cigarette smoking and famine. Both have shown associations with outcomes to grandchildren. However, it is not clear whether studies of the intergenerational associations with ancestral famine were due to severe lack of nutrition or to the associated stress of the experience (which was often experienced in wartime). Here we have shown, possibly for the first time, that the trauma of a grandparent experiencing the death of a parent during their childhood was associated with their grandchildren having an increased risk of developing asthma. This may not indicate stress but rather that the death of a father would have often been associated with a loss of income to the family and thence poorer environmental exposures or inadequate dietary intake.

One possible explanation for intergenerational associations could involve the fact that offspring of mothers who themselves smoke in pregnancy are more at risk of developing asthma (Zacharasiewicz, 2016). However, if maternal smoking did play a role in the associations with the maternal grandmother smoking in pregnancy, it would presumably be as a mediator, not a confounder. In our earlier studies of grandmaternal smoking we looked at individuals whose maternal grandmother had smoked in pregnancy, but whose mother had not (results reviewed in Golding et al., 2021). For asthma at age 7, results were similar to those shown in this paper, with increased AOR for paternal but not maternal grandmother smoking in pregnancy (Miller et al., 2014). The current results below for asthma at age 7 show that omitting children whose mothers smoked in pregnancy made little difference to the unadjusted ORs although confidence intervals were slightly wider due to a reduction in numbers caused by omitting mothers who had smoked in pregnancy.

$$MGM+OR=1.16\;\left[95\%CI\;1.03,1.30\right]$$

$$MGM+M-OR=1.14\;\left[95\%CI\;1.00,1.30\right]$$

Where + indicates smoking during pregnancy, - indicates did not smoke during pregnancy, MGM is the maternal grandmother and M indicates the mother.

Nevertheless, even if maternal smoking in pregnancy was a mediator for the maternal grandmother smoking associations, this would not explain the associations we found with paternal grandmother smoking, nor the sex specific effects, which are the main findings in the present paper.

### Strengths and limitations

The major strengths of the study lie in the following: i) the information was collected from the study parents (labelled M and F in Figure 1) with no indication that the data were being collected to assess grandparental effects on the health of the study children. Consequently, the data were unlikely to suffer from recall bias. ii) The measures of stress used (death of a parent) were categorical easily identified factors—and not likely to have been biased in any way. iii) The data were available to determine intergenerational associations with asthma in young children (aged 7 years) and then in early adulthood (aged 22 years). iv) By looking at two distinct generations we were able to assess whether associations, for example, with death of a parent or with adolescent smoking, were consistent within cohorts.

There are a number of limitations: i) As with all longitudinal studies, considerable attrition occurred over time. However, although the study began with over 14,000 pregnancies, the study is still in touch with approximately 8000 of the F3 generation. However, the numbers with data available for these analyses were relatively small—even though they are probably the largest available at the present time. ii) The study began in 1990–1 when the proportion of births to ethnic minorities in the study area was small (∼6%); consequently, there was no chance of analysing by ethnic minority. iii) We only used participant report of a history of clinically recognised asthma; however, a diagnosis of asthma is often made for a wide variety of respiratory symptoms without objective testing and so may include alternative or co-existing diagnoses such as hyperventilation, breathing pattern disorders, obesity related breathlessness—all of which may mimic asthma. iv) we had no knowledge of the type of cigarettes smoked by the grandparents, nor the frequency with which they had smoked during adolescence or pregnancy. v) Although we accounted for the demographic variables that appeared likely to be confounders, we acknowledge that residual confounding may be an issue here. vi) The number of analyses undertaken provides the usual problems with multiple testing: we showed that there were considerably more sex-specific results at *p* < 0.05 than would be expected by chance; these would have been ignored if we had stuck to the Bonferroni adjusted threshold of *p* < 0.001 which would have resulted in a conclusion of no associations. We feel that by publishing all AORs at *p* < 0.05, others will be able to compare and thence strengthen these results.

### Conclusion

We undertook this study to answer two main questions:

a) Whether our earlier result in the ALSPAC generation F3 which showed an association between paternal grandmother smoking in pregnancy and asthma in the grandchild was consistent over time and between generations. We have shown that indeed the association was consistent over time (by comparing results between the relationships for 7- and 22-year- olds), and between the F2 and F3 generations.
b) To determine whether there were any associations between smoking during the adolescent period of the grandparents, or between stressors in childhood and asthma in the grandchildren We have shown that smoking in adolescence of the paternal grandmother was associated with grandchild’s asthma in the F3 cohort. In addition, we have shown that death of a father during a paternal grandmother’s childhood was associated with asthma in grandchildren in the F3 generation.

There were additional minor questions concerning whether there were sex effects or whether the maternal or paternal line was most important. We found some evidence for differential associations with the sex of the grandchild, but on average it has been exposures to the paternal rather than maternal grandparental line that have been most apparent.

We are aware that these results need to be repeated in other longitudinal intergenerational studies but hope that they will spur other cohorts to collect relevant intergenerational data, particularly information concerning paternal grandparents. Similar intergenerational findings are often thought to be due to epigenetic effects, with DNA methylation being the mechanism most often quoted. Elsewhere, using blood samples from these F3 grandchildren, Watkins et al. (2022) have identified different methylation markers associated with smoking in pregnancy of the F1 maternal as well as the F1 paternal grandmothers. However, it is premature to speculate further on possible mechanisms for our findings until confirmatory evidence is available from other cohorts.

## Data Availability

The data analyzed in this study is subject to the following licenses/restrictions: ALSPAC data access is through a system of managed open access. The steps below highlight how to apply for access to the data included in this paper and all other ALSPAC data. Please read the ALSPAC access policy (http://www.bristol.ac.uk/media-library/sites/alspac/documents/researchers/data-access/ALSPAC_Access_Policy.pdf) which describes the process of accessing the data and biological samples in detail, and outlines the costs associated with doing so. 1. You may also find it useful to browse our fully searchable research proposals database (https://proposals.epi.bristol.ac.uk/), which lists all research projects that have been approved since April 2011. 2. Please submit your research proposal (https://proposals.epi.bristol.ac.uk/) for consideration by the ALSPAC Executive Committee using the online process. You will receive a response within 10 working days to advise you whether your proposal has been approved. If you have any questions about accessing data, please email: alspac-data@bristol.ac.uk (data) or bbl-info@bristol.ac.uk (samples). The ALSPAC data management plan (http://www.bristol.ac.uk/media-library/sites/alspac/documents/researchers/data-access/alspac-data-management-plan.pdf) describes in detail the policy regarding data sharing, which is through a system of managed open access. . Requests to access these datasets should be directed to alspac-exec@bristol.ac.uk.
