# Supplementary material for: A history of asthma may be associated with grandparents’ exposures to stress and cigarette smoking
Source: Front Toxicol. 2023 Sep 22;5:1253442. doi: 10.3389/ftox.2023.1253442 (PMC10556739; doi:10.3389/ftox.2023.1253442)
Supplement: Supplementary file 1 [file Table1.DOCX]

**Supplementary tables**

**Supplementary Table 1. Numbers of granddaughters and grandsons with demographic data on their grandparents’ childhoods as well as their own history of asthma at age 7 years.**

| ***Granddaughters*** | | | | |
| --- | --- | --- | --- | --- |
| *Demographic Features* | MGM | MGF | PGM | PGF |
| Year of birth | 3506 | 3299 | 2646 | 2027 |
| Place of birth | 1907 | 1837 | 032 | 908 |
| Education level | 2852 | 2692 | 2334 | 2330 |
| Social class | 2083 | 3120 | 1628 | 2832 |
| *Environmental exposures* |  |  |  |  |
| Traumatic events | 1830 | 1674 | 876 | 873 |
| Smoking in adolescence | 1687 | 1471 | 773 | 678 |
| Smoking in pregnancy | 3708 | X | 3045 | X |
| ***Grandsons*** | | | | |
| *Demographic Features* |  |  |  |  |
| Year of birth | 3722 | 3491 | 3192 | 2657 |
| Place of birth | 1736 | 1682 | 813 | 783 |
| Education level | 3077 | 2913 | 2508 | 2509 |
| Social class | 2231 | 3310 | 1686 | 2961 |
| *Environmental exposures* |  |  |  |  |
| Traumatic events | 1661 | 1534 | 762 | 710 |
| Smoking in adolescence | 1539 | 1354 | 663 | 564 |
| Smoking in pregnancy | 3919 | X | 3186 | X |

X = not relevant

**Supplementary Table 2a. Relationships (P-values) between grandchildren’s experience of a history of asthma by age 7 and the potential demographical confounders of their grandparents.** Associations at P<0.05 are in bold.

| ***Demographic***  ***Features*** | **MGM** | **MGF** | **PGM** | **PGF** |
| --- | --- | --- | --- | --- |
| Year of birth | **<.0001** | **<.0001** | **<.001** | **<.001** |
| Resident in England at birth | .076 | .531 | .468 | .854 |
| Education level | .424 | **.029** | **.036** | .050 |
| Age at birth of parent | **.012** | **<.001** | **.039** | **.036** |
| Social class | **.029** | .268 | .903 | **.019** |

**Supplementary Table 2b. Relationships (P-values) between grandchildren’s experience of a history of asthma by age 22 and the potential demographical confounders of their grandparents.** Associations at P<0.05 are in bold.

| ***Demographic***  ***Features*** | **MGM** | **MGF** | **PGM** | **PGF** |
| --- | --- | --- | --- | --- |
| Year of birth | **.025** | **.011** | .959 | .789 |
| Resident in England at birth | .084 | .705 | .792 | .369 |
| Education level | .709 | .828 | .441 | .900 |
| Age at birth of parent | .230 | .074 | .831 | .690 |
| Social class | .650 | .306 | .644 | .820 |

**Supplementary Table 2c. Relationships (P-values) between granddaughter’s experience of a history of asthma by age 14-47 and the potential demographical confounders of her grandparents.** Associations at P<0.05 are in bold.

| ***Demographic***  ***Features*** | **MGM**  **=**  **MGMM** | **MGF**  **=**  **MGMF** | **PGM**  **=**  **MGFM** | **PGF**  **=**  **MGFF** |
| --- | --- | --- | --- | --- |
| Year of birth | .593 | .863 | .134 | .092 |
| Resident in England at birth | .923 | .387 | .650 | .518 |
| Age at birth of parent | .986 | .823 | **.011** | **.006** |
| Social class | .898 | .199 | .636 | .303 |

**Supplementary Table 2d. Relationships (P-values) between grandson’s experience of a history of asthma by age 15-65 and the potential demographical confounders of their grandparents.**

| ***Demographic***  ***Features*** | **MGM**  **=**  **MGMM** | **MGF**  **=**  **MGMF** | **PGM**  **=**  **PGFM** | **PGF**  **=**  **PGFF** |
| --- | --- | --- | --- | --- |
| Year of birth | .302 | .615 | .295 | .101 |
| Resident in England at birth | .594 | .101 | .246 | .645 |
| Age at birth of parent | .115 | .737 | .609 | .101 |
| Social class | .906 | .570 | .900 | .275 |

**Supplementary Table 3a. The adjusted odds ratios concerning grandparental exposures associated with asthma in the F3 grandsons and granddaughters at age 7,** Associations at P<0.05 are in bold.

| **Grandparent** | | **Childhood**  **Exposures** | **Grandsons** | | **Granddaughters** | |
| --- | --- | --- | --- | --- | --- | --- |
|  |  |  | **AOR [95%CI]** | **P** | **AOR [95%CI}** | **P** |
| MGM |  | Mother died | 1.13[ .68,1.88] | .650 | **1.87[1.09, 3.22]** | **.023** |
|  |  | Father died | 1.45[.93, 2.160] | .069 | 1.11[.70, 1.75] | .664 |
|  |  | Smoked <17y | 1.08[.69,1.69] | .725 | 1.43[.93, 2.19] | .102 |
|  |  | Smoked in  Pregnancy | .85[.59, 1.21] | .365 | 1.05[.73, 1.50] | .785 |
|  |  |  |  |  |  |  |
| MGF |  | Mother died | 1.23[.71, 2.10] | .467 | 1.39[.84, 2.29] | .202 |
|  |  | Father died | .89[.56, 1.40] | .620 | .95[.59, 1.51] | .815 |
|  |  | Smoked <17y | .96[.66, 1.40] | .836 | .95[.59, 1.50] | .859 |
|  |  |  |  |  |  |  |
| PGM |  | Mother died | 1.85[1.08, 3.69] | .081 | .78[.32, 1.92] | .586 |
|  |  | Father died | 1.36[.71, 2.62] | .357 | **1.85[1.02, 3.37]** | **.044** |
|  |  | Smoked <17y* | **2.89[1.57, 5.35]** | **.001** | 1.18[.59, 2.39] | .635 |
|  |  | Smoked in  Pregnancy | **1.30[1.02, 1.64]** | **.035** | **1.33[1.02, 1.73]** | **.032** |
|  |  |  |  |  |  |  |
| PGF |  | Mother died | 1.22[.54, 2.64] | .635 | 1.56[.79, 3.05] | .206 |
|  |  | Father died | 1.43[.72, 2.73] | .281 | 1.59[.88, 2,86] | .121 |
|  |  | Smoked <17y | 1.42[.77, 2.62] | ,257 | 1.49[.82, 2.72] | .191 |
|  |  |  |  |  |  |  |

*sex difference

**Supplementary Table 3b. The adjusted odds ratios concerning grandparental exposures associated with asthma in the F3 grandsons and granddaughters at age 22,** Associations at P<0.05 are in bold.

| **Grandparent** | | **Childhood**  **Exposures** | **Grandsons** | | **Granddaughters** | |
| --- | --- | --- | --- | --- | --- | --- |
|  |  |  | **AOR [95%CI]** | **P** | **AOR [95%CI]** | **P** |
| MGM |  | Mother died | 1.06[.63, 1.77] | .841 | 1.25[.82, 1.92] | .305 |
|  |  | Father died | .87[.56,1.35] | .537 | 1.09[.65, 1.33] | .629 |
|  |  | Smoked <17y | .69[.44,1.08] | .106 | .92[.65, 1.22] | .692 |
|  |  | Smoked in  pregnancy | .95[.66, 1.37] | .787 | .92[.70, 1.22] | .565 |
|  |  |  |  |  |  |  |
| MGF |  | Mother died* | **.44[.23, .84]** | **.013** | .89[.53, 1.54] | .667 |
|  |  | Father died | 1.03[.55,1.91] | .939 | **.58[.35, .96]** | **.035** |
|  |  | Smoked <17y | .75[.46,1.21] | .238 | .81[.56,1.16] | .246 |
|  |  |  |  |  |  |  |
| PGM |  | Mother died | 1.25[.62,1.51] | .531 | 1.01[.60,1.70] | .961 |
|  |  | Father died | .87[.49,1.52] | .613 | 1.21[.35, 1.43] | .372 |
|  |  | Smoked <17y | .76[.41,1.42] | .387 | 1.21[.75, 1.48] | .438 |
|  |  | Smoked in  Pregnancy* | .90[.67,1.20] | .457 | 1.20[.97, 1.33] | .099 |
|  |  |  |  |  |  |  |
| PGF |  | Mother died | 1.29[.64,2.59] | .477 | .91[.55, 1.49] | .696 |
|  |  | Father died | .83[.48,1.43] | .501 | 1.18[.76, 1.84] | .453 |
|  |  | Smoked <17y | .89[.53,1.49] | .652 | 1.34[.92, 1.97] | .130 |
|  |  |  |  |  |  |  |

*Sex differences
